# Architecture and Development Framework for a Web-Based Risk Assessment and Management Platform Developed on WordPress to Address Opioid Overdose

**DOI:** 10.2196/49759

**Published:** 2024-03-11

**Authors:** Alireza Kazemi, Marisha Boyd, Fiona Choi, Andy Man Yeung Tai, Vivian WL Tsang, Tam To, Jane Kim, Kerry Jang, Farhud Shams, Stefanie Schreiter, Maurice Cabanis, Reinhard Michael Krausz

**Affiliations:** 1 Institute of Mental Health Department of Psychiatry University of British Columbia Vancouver, BC Canada; 2 Department of Psychiatry and Neurosciences Charité Campus Mitte Institution: Charité – Universitätsmedizin Berlin, corporate member of Freie Universität Berlin and Humboldt-Universität zu Berlin Berlin Germany; 3 Hospital for Addiction and Addictive Behavior Center of Mental Health Klinikum Stuttgart Esttutgarth Germany

**Keywords:** software designs, risks management, risk assessments, opioid overdose, crisis intervention, substance related disorders

## Abstract

The number of overdose-related fatalities continues to reach historic levels across Canada, despite ongoing efforts by authorities. To reduce mortality, a clinical trajectory ranging from preventative measures to crisis intervention, skill training to treatment, and risk assessment to risk management needs to be supported. The web-based Risk Assessment and Management Platform (RAMP) was developed to realize this concept and to empower people who use drugs through an integrated tool that allows them to better understand and manage their risk of overdose. This paper outlines the architecture and development of RAMP, which is built on the WordPress platform. WordPress components are mapped onto a 3-tier architecture that consists of presentation, application, and database layers. The architecture facilitates the development of a modular software that includes several features that are independent in functionality but interact with each other in an integrated platform. The relatively low coupling and high coherence of the features may reduce the cost of maintenance and increase flexibility of future developments. RAMP’s architecture comprises a user interface, conceptual framework, and backend layers. The RAMP front end effectively uses some of the WordPress’ features such as HTML5, CSS, and JavaScript to create a mobile, friendly, and scalable user interface. The RAMP backend uses several standard and custom WordPress plug-ins to support risk assessment and monitoring, with the goal of mitigating the impacts and eliminating risks together. A rule-based decision support system has been hard-coded to suggest relevant modules and goals to complement each user’s lifestyle and goals based on their risk assessment. Finally, the backend uses the MySQL database management system and communicates with the RAMP framework layer via the data access layer to facilitate a timely and secure handling of information. Overall, RAMP is a modular system developed to identify and manage the risk of opioid overdose in the population of people who use drugs. Its modular design uses the WordPress architecture to efficiently communicate between layers and provide a base for external plug-ins. There is potential for the current system to adopt and address other related fields such as suicide, anxiety, and trauma. Broader implementation will support this concept and lead to the next level of functionality.

## Introduction

### The Clinical Problem

In 2016, the British Columbia government declared a state of emergency in response to the increasing number of drug-related overdose fatalities [1]. Despite this measure, more than 14,000 British Columbians have since died due to high potent opioids, and the province has seen a >2-fold increase in the number of overdose deaths, from 20.5 per 100,000 individuals in 2016 to 46 per 100,000 individuals in 2023 [2]. High-risk substance use and overdose risk is often a reflection of complex concurrent mental and physical conditions, which are critical for understanding the risk constellation as well as overdose risk management [3,4]. Integration of informed approaches to address severe and early traumatic experiences, suicidality, and mental illness is necessary for a solution aimed at increasing the chance of survival and recovery among people who use drugs [5]. Web-based interventions have demonstrated effectiveness in improving health outcomes and reducing barriers for people who use drugs who are seeking support [6,7].

### A Modular Architecture

Modular architecture is a model of designing web-based psychotherapy platforms and interventions [8,9]. A modular design implies that the content presented can be divided into multiple smaller pieces that can be completed independently or as a set [9]. The strengths of modular systems include the ability for users to focus on 1 area at a time and ease in replicating modules following a common structure. Therefore, this approach allows for the inclusion of new clinical trajectories beyond substance use and overdose for a significantly lower price. It will also reduce the cost of maintenance. This architecture lends itself well to web-based interventions for substance use because sections, topics, and features can work both independently and together to provide care to a unique population with diverse needs. Chronic conditions such as substance use disorder need to be seen as a long-term trajectory—a dynamic and often changing process, which requires a range of tools to adapt to the needs of users.

An ideal web-based platform addressing substance use would create an environment that provides tools to assess risks, gain deeper knowledge of risks, prevent deterioration and crises, and manage specific behavioral factors and symptoms. The tasks for the development team would be to add content and functionalities to make the system engaging and interactive. Our vision is a platform that can become a long-term tool providing an engaging learning environment where user choices and outcomes influence the trajectory and functionality of the platform. Tools such as machine learning and embedded trials would allow for a systematic internal evaluation of user choices as well as better referrals and decision support tools. Users can adapt the content and functionalities to their needs, add content and their own experiences, and provide feedback to become active drivers of the system.

### Risk Assessment and Management Platform

**Figure 1:**
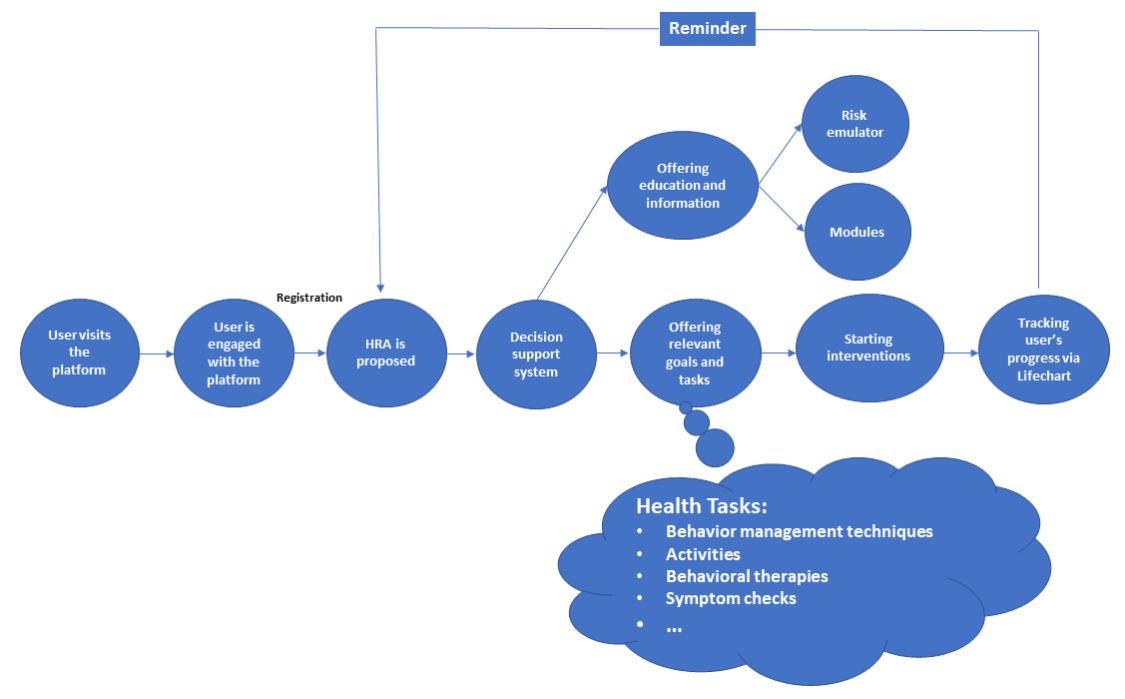
Interaction among different features of the Risk Assessment and Management Platform to identify and manage the risk of overdose. HRA: High-Risk Assessment.

The Risk Assessment and Management Platform (RAMP) was developed by the Addictions and Concurrent Disorders Research Group at the University of British Columbia, supported by funding from Health Canada. The goal of this web-based platform is to provide a low-barrier tool that educates and empowers users to make informed decisions for their own recovery journey. The platform consists of self-assessments, educational and therapeutic modules, and tools to help users plan and monitor their risk of overdose, for example, “Goal setting” and “Lifechart.” Interactions among RAMP features to identify and manage the risk of overdose are shown in Figure 1.

Users must create a free account in order to use the full functionalities of RAMP, including having their scores and data tracked over time. New users have the option to explore some selected features, such as modules and “Art with a Meaning” before they register. This allows users to better understand the system and feel comfortable with it enough to create an account. It also enables public access to modules, which, on its own, will have significant psychotherapeutic benefits for users.

Upon registration, the High-Risk Assessment (HRA) is proposed to the user. The HRA is a screening tool developed by the Addictions and Concurrent Disorders Research Group to identify the risk of overdose among people who use drugs through a rule-based algorithm. Development of the HRA will be explained in a separate publication.

Based on the results of each user’s HRA score, a clinical decision support system suggests the most relevant modules and goals to the user. The user may comply with the recommendations or choose to work on additional or substitute modules of their choice. The modules will help the user to gain knowledge about their risky behaviors and how to prevent or mitigate those risks. The Goals setting function will allow the user to make their risk mitigation plan and monitor their progress through the Lifechart feature. An email notification is sent to the user to inform them about their progress and to encourage them to continue interacting with the software by reminding them about their recent usage and how they can resume.

The core concepts and theoretical frameworks of RAMP will be presented in a series of publications. Topics will include the clinical concept underlying RAMP, development of the HRA, development of modules, and user interface evaluation. This paper is focused on the design and architecture of RAMP. The modular architecture of RAMP uses WordPress’ multilayer architecture, which lends itself well to the dynamic and customizable application design.

### Design of RAMP

#### Overview

RAMP is a web application developed using the WordPress content management system (CMS). Therefore, description of the architecture of WordPress is essential to describe RAMP’s design and architecture.

WordPress is built on a PHP and MySQL architecture [10]. It uses a multilayer architecture to provide a flexible platform for building and customizing websites [11]. The purpose of a layered architecture is to organize the components of an application into horizontal logical layers and physical tiers. A layer is a logical unit that separates a specific role and responsibility within an application. Each layer manages its own software dependencies. A tier is a physical unit where the code runs, for example, a web server or a database. Scalability, maintainability, and resiliency are increased when tiers are physically separated; however, latency increases due to additional network communication.

Though WordPress does not strictly follow the traditional 3-tier architecture to separate the presentation, application, and database tiers, the WordPress components that are used to develop web applications can be mapped on a 3-tier design model [12,13].

#### Presentation Tier

The presentation tier is responsible for showing the content and functionality to the user, using HTML, CSS, and JavaScript. HTML provides a way to create and structure the elements of a web page, such as headings, paragraphs, images, links, etc. CSS is a powerful tool for customizing the appearance and layout of WordPress websites. JavaScript is a scripting language that is used to add interactivity, dynamic behaviors, and effects such as animations, interactive forms, and user interface elements such as sliders and pop-up menus to web pages.

#### WordPress Themes

Themes control the appearance of a WordPress website. They include templates, CSS files, and other components that determine how the site is displayed.

#### Application Tier

##### Overview

The application tier in WordPress refers to the core PHP code that provides the underlying functionality and controls the flow of data between the presentation and database tiers. The application tier includes the WordPress core, plug-ins, and custom code that interacts with the database to store and retrieve data.

Plug-ins are add-ons that extend the functionality of WordPress. They can provide additional features such as contact forms, e-commerce functionality, etc.

The application tier is responsible for implementing the business logic as well as accessing the data stored in the database through the data access layer.

##### Business Logic Layer

The business layer in WordPress architecture refers to the layer that handles the business logic and processes in a WordPress website. This layer is responsible for performing tasks such as data validation, calculations, and decision-making.

In the context of WordPress, the business layer can be implemented in a number of ways, including custom plug-ins, custom code within the theme, or through the use of third-party plug-ins that provide specific functionality.

The business layer acts as the intermediary between the presentation and database tiers. It takes input from the presentation layer, performs the necessary business logic, and then communicates with the database tier to retrieve or store data. This architecture allows for greater flexibility and maintainability of WordPress products, as well as the ability to easily modify and extend the functionality of the website without affecting other tiers.

##### Data Access Layer

The data access layer in WordPress architecture refers to the layer responsible for accessing and manipulating data stored in the database. This layer is responsible for retrieving data from the database, performing operations on the data, and storing the results back in the database.

In WordPress, the data access layer is typically implemented using the WordPress database application programing interface (API), which provides a set of functions and methods for interacting with the database. These functions and methods allow developers to perform common database operations, such as inserting, updating, and retrieving data, in a consistent and efficient manner.

##### Database Tier

The database tier in WordPress is where all the data are stored, including posts, pages, users, and metadata. WordPress uses a Relational Database Management System, MySQL, to store this information, and it is accessed by the application tier to retrieve and manipulate data.

The database layer in WordPress works by creating tables and storing data in those tables. Each table contains rows and columns that represent the different data types stored in the database. For example, WordPress has separate tables for posts, users, comments, etc.

When a user interacts with a WordPress site, the database layer retrieves data from the appropriate table and returns it to the application tier. The data can then be displayed on the user interface of the site or being used to perform various operations in the backend.

In conclusion, the database layer in WordPress applications is a critical component that provides a structured way to store and retrieve data and plays an important role in the overall architecture of a WordPress site.

Overall, the architecture of WordPress promotes modularity. This modular design approach allows for easier customization, scalability, and maintainability of software products built on the WordPress platform. Developers can create independent modules that can be combined or modified without significant impact on other components, facilitating the creation of flexible and extensible software systems.

RAMP software design architecture follows the WordPress multilayer architecture.

#### Ethical Considerations

The RAMP HRA pilot project has undergone research ethics review and received ethics approval from the University of British Columbia Behavioural Research Ethics Board (H19-02231). Collection of data on RAMP is limited to only those necessary for the research objectives. Participant identities are kept confidential, and the research protocol is aligned with the Tri-Council Policy Statement on Ethical Conduct for Research Involving Humans (TCPS2). Informed consent forms include information about data storage, data security, data sharing, privacy protections, legal limits to confidentiality, and contact information of the University of British Columbia Behavioural Research Ethics Board. It clearly delineates how the data will be used and shared, and the process to report participant concerns.

Upon registration, the system stores their registered email IDs, names, last names, usernames, passwords (encrypted), login times, and IP addresses. Data are stored on secure cloud servers, and security features such as reCAPTCHA and temporary login blocks are used to minimize the risk of brute force attacks. Upon login, their interactions with the system are stored and tracked. This includes assessments results, symptoms check, completed modules, goals, journal diaries, and achievements. Data are not shared with third parties unless for maintenance, software improvements, or legal obligations. The overall data governance plan of the project is modeled after the highest local, federal, and international standards of privacy, data management, and data sharing.

### System Description

#### Overview

RAMP is designed as an engaging, learning, and open access web application that empowers users to manage their risk of overdose and concurrent mental health disorders. RAMP is a modular software that includes several features and functionalities.

**Figure 2:**
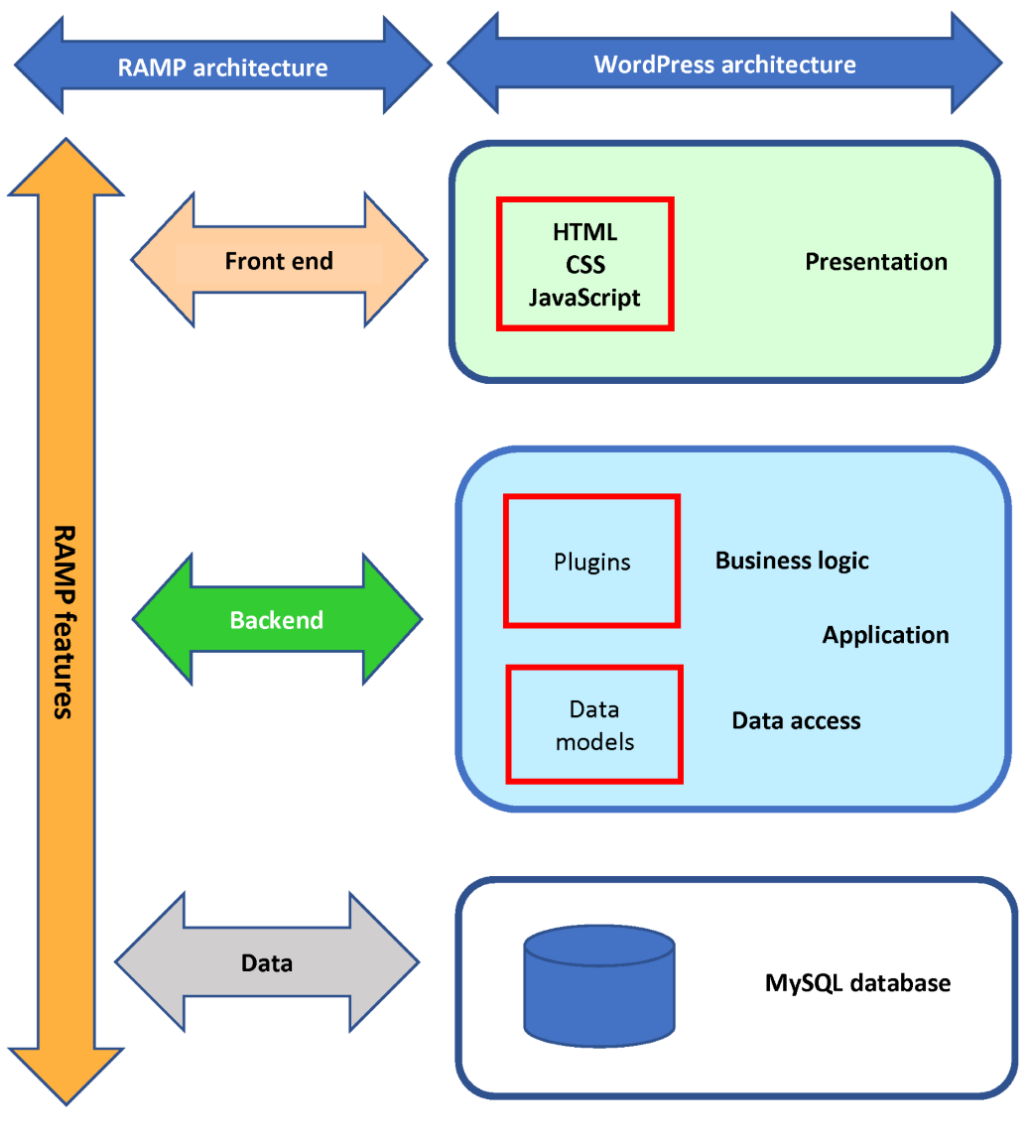
Mapping RAMP features on a 3-tier software architecture model that uses WordPress components. RAMP: Risk Assessment and Management Platform.

The architecture and design of RAMP features is mapped on a 3-tier architecture model that uses WordPress components. Figure 2 demonstrates the mapping.

#### Data Tier

The Data Tier uses relational databases on the MYSQL 5.7.30 database management system. RAMP stores and retrieves various types of data including text, video, audio, and images, which can become bulky over time. The data layer is managed by the WordPress database tier, which provides secure and efficient methods to store, retrieve, and organize data. It uses indexing, caching, lazy loading, query optimization, and other techniques to improve its efficiency.

#### The Backend

**Figure 3:**
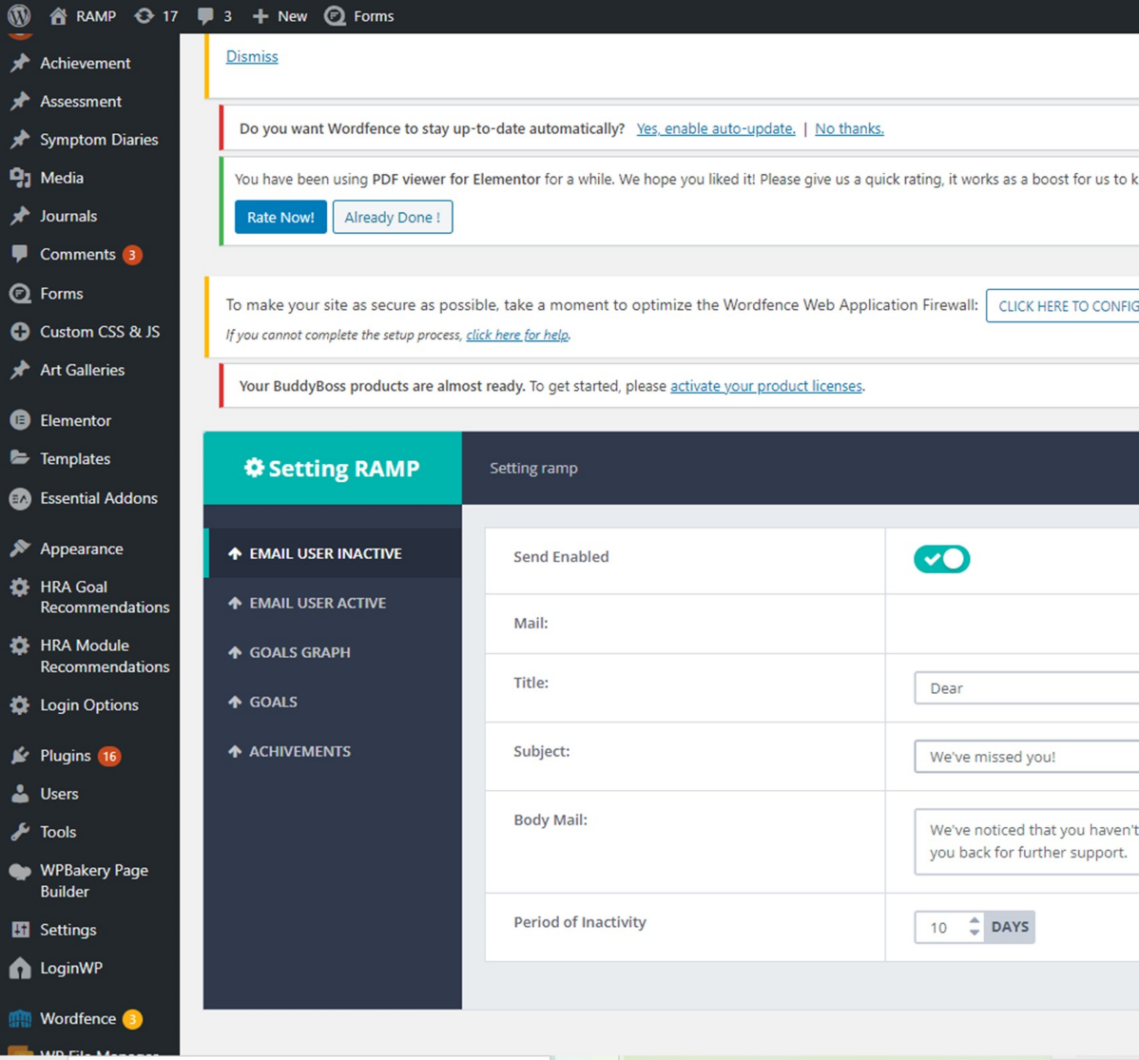
RAMP’s administrative panel on WordPress (accessible to administrators only).

The backend uses the WordPress 5.4 development framework. The system is a web application software compatible with most of the standard browsers such as Chrome, Safari, and Edge, and is responsive to mobile apps. The system’s administrative panel allows the administrator to add new reports, reorganize some user interface elements, and define new assessments (Figure 3). This panel also allows the administrator to monitor users’ activities and progresses and add new modules and other content. Certain key performance indicators can be found on the administrative panel.

The RAMP backend also uses some standard and custom plug-ins. Some examples of the plug-ins that RAMP is using are listed below:

- Disable XML-RPC-API: a lightweight plug-in to disable XML-RPC API, Pingbacks, and Trackbacks for faster and more secure browsing of a website.
- Duplicate Page: a plug-in to duplicate posts, pages, and custom posts with a single click.
- LoginWP: a plug-in to redirect users to different URLs based on their role, capability, and more.
- Quform: a plug-in to create powerful and engaging quizzes, tests, and examinations within minutes, and to build an unlimited number of quizzes and questions.
- WP File Manager: a plug-in that manages WP files.
- WP SMTP: a plug-in that helps developers to send emails via SMTP instead of the PHP mail function. This plug-in is particularly useful to send email reminders to the user, which would less likely end up in junk mail; support superior authentication and encryption methods; and is more compatible with other WordPress plug-ins.

There are custom-built plug-ins created for different RAMP features such as Achievements, Assessments, Symptom Diary Forms, Journal, and Lifechart to enhance user experience.

The inference engine of a rule-based decision support system is custom-coded in the business layer. User responses to the HRA are used to determine relevant modules and goals, and to suggest them via the user interface.

The data access layer in RAMP uses the wp-config file to configure the database through different parameters. These parameters allow consistent and secure access to all tables of the RAMP database.

#### Conceptual Framework

**Figure 4:**
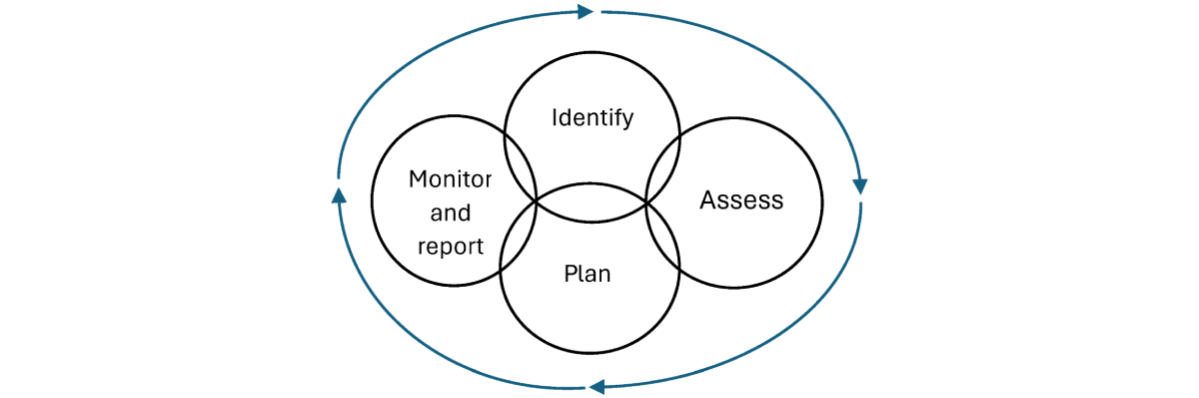
Risk management process.

RAMP’s conceptual framework is part of the backend and is currently tailored to substance use but can be modified to manage the risk of other mental health conditions such as suicide and trauma. The principles are in line with the core concept of the risk management process, which includes identifying and assessing risks, making a plan, and monitoring the plan to be able to mitigate or eliminate the risk as needed (Figure 4).

#### The Front End

RAMP front end uses WordPress presentation layer components such as themes, HTML5, CSS, and JavaScript to communicate with the user and provide a pleasant experience when showing content.

RAMP uses BuddyBoss as its core theme. However, a subtheme was derived and customized from BuddyBoss to enhance user experience and use consistent visual themes across RAMP’s branding.

Using HTML5 in RAMP has created more modern and effective web pages for the system with better accessibility, improved search engine optimization, higher performance, and mobile friendliness. Additionally, pages have been further fine-tuned by the developers for a better mobile experience. HTML5 has also allowed the developers to embed audio and video content directly into the web pages, eliminating the need to rely on third-party plug-ins such as Adobe Flash. RAMP is also using native lazy loading of images and script execution in parallel, which can improve the performance of web pages and reduce their load times.

RAMP uses CSS effectively to create websites that are visually appealing, flexible, responsive to mobile devices, and easy to use.

#### Interaction With External Systems

RAMP uses different WordPress plug-ins and features to interact with different external systems. YouTube videos in the modules are embedded into Elementor, a popular visual page builder plug-in for WordPress that allows users to create custom designs for their website and ensures the correct publication of the videos.

The mental health resource map is another example of an external system that is integrated into RAMP. The development team used Google My Maps to pin the location of mental health services, including clinics, youth services, and urgent care centers, onto a Google map. This software enables users to view services and facilities in their area, find their locations, hours of operation, contact information, and more on a Google map in a layered format. The system embeds this software using a simple script method by adding its link to RAMP.

The development team has also been working on a parallel project to create a machine learning model that would predict the risk of fatal overdose based on a comprehensive data set called the British Columbia Provincial Overdose Cohort curated by the British Columbia Centre for Disease Control. The model will subsequently be embedded into an API, which allows the predictive model to be used as a stand-alone tool. The aim is to use the developed predictive model in RAMP using a service-oriented architecture, where RAMP calls upon the predictive model’s API using WordPress REST API plug-in to send user-specific risk factors to the model. The model will then process the overall risk of fatal overdose based on user inputs, determine the likelihood of overdose, and send the results back to RAMP. The RAMP interface will display the results and provide tailored recommendations based on each individual’s risk factors.

## Discussion

### Background

The aim of RAMP is to empower people who use drugs to become more aware of their overdose risk and to provide a tool that promotes agency in helping them manage their risk factors. Although risk management is broad and may include several activities, the core concepts include key steps to identify, plan, and monitor risks, as well as tools to execute the plan to eliminate or mitigate the impact of these risks [14].

RAMP is a web application. In contrast to native apps, web applications have certain advantages that RAMP benefits from:

- Cross-platform compatibility and being installation-free: RAMP can run on any device with a web browser, which means that it is platform-independent and can be accessed from a wide range of devices, including desktops, laptops, tablets, and smartphones without the need to install the software on the device. In contrast, native apps are developed for a specific platform, such as iOS or Android, and need to be downloaded and installed on the user's device. This is helpful when considering the population of people who use drugs, who might not have consistent access to a smartphone but can access public or shared computers [15].
- Easier maintenance: RAMP maintenance and updating is easier and less costly than native apps, as changes can be made on the server side, and users are not required to download updates. This can save time and resources for developers and ensure that all users are using the most up-to-date version of RAMP.
- Lower development costs: developing a web application typically requires fewer resources than developing a native app, as there is no need to develop separate versions for different platforms such as iOS and Android. This can result in lower development costs and less time to deploy the application.

While web applications offer certain benefits, native apps offer advantages such as better performance, native features, push notifications, and access to device-specific capabilities. Web applications always need an active internet connection to the server, but native apps may not need constant access to the internet if there is no need to communicate with a centralized server on a continuous basis.

RAMP features effectively use the WordPress components and their multilayer architecture to support the user to make informed decisions. One example is the design of HRA, a 29-question screening tool that helps users identify several overdose risk factors across different domains.

The integration of a dynamic color-coded heat map on the user interface of the presentation tier, featuring a sliding score bar that slides as the questions are answered, assists the user in comprehending the relative significance of individual factors and whether they represent risks or protective factors. Upon responding to each question, the inference engine of a rule-based decision support system will use the responses that are stored in the RAMP database layer to determine and suggest the most relevant modules and goals that are critical to the management of the most pressing risks. The use of these features across layers empowers the user to accurately identify their risks, gain knowledge of such risks, establish relevant goals, and monitor progress—all through the use of RAMP.

RAMP’s modular design allows novice users to follow the decision support system recommendations and create relevant goals and educational plans, while providing more freedom to experienced users to focus on specific areas that are most related to their personal risk of overdose and lifestyle. This design is important for the risk planning phase. When the user executes the plan and performs the given tasks, the system provides rewards to further engage the user.

### Strategies to Enhance User Engagement

Reward systems are one of the most common gamification strategies designed to encourage users. The system awards badges as the user makes progress. This provides reinforcement as an individual continues their recovery journey. There is broad interest in the use of digital badges to enhance learners' motivation [16]. These web-enabled tokens of learning and accomplishment have the potential to induce excitement and elicit powerful forms of engagement and learning. Badges are one of the most powerful gaming elements that are widely used in digital health products to motivate users to keep interacting with the system [17]. This strategy is particularly important for the population of substance users where retention and long-term interaction might be difficult [18,19].

RAMP does not support on-screen or push notifications. Instead, the system uses reliable SMTP email notifications regularly to inform users about their progress and motivate them to continue their interaction with the system. Notifications are an important gamification tool to promote engagement with the platform [20]. The frequency and content of RAMP notifications is different for active and inactive users. Active users receive more frequent notifications with suggestions regarding next steps they might benefit from based on their current patterns of use, while inactive users may receive positive reinforcement notifications and encouragements to start interacting with the system or continue where they have stopped. Previous studies have shown that tailoring notifications to each user is an effective way of boosting the use of web-based health tools [21].

### Application of RAMP to Other Disorders and Diseases

RAMP content is currently developed to support those struggling with substance use and who are at risk of overdose. However, the tool can also be used to empower patients in other related domains such as navigating trauma, managing anxiety, and preventing self-harm and suicide by adding relevant assessments, modules, and goals. Through the development of proper content, the same logic can be applied to other mental health domains without a need to customize or redevelop the features, which maximizes the reusability of the developed system. Furthermore, RAMP features are fairly independent but interact with each other based on the business logic and through interfaces. This is in line with the principles of software engineering in which subsystems of a modular software should have high internal cohesion and low coupling [22].

### Limitations

The knowledge of the authors about the application of WordPress plug-ins and add-ons is limited to RAMP. RAMP is a web application; therefore, general limitations of web applications such as requiring continuous internet connection, etc, are also applicable to the present software.

RAMP is developed on the WordPress CMS. Security and performance of the software are limited to the WordPress development framework. Although many successful and secure applications are developed on the same CMS platform, there is news about possible security exploitations of the websites developed on WordPress such as being infected by malwares, backdoors, or being hacked [23,24]. The RAMP development team has attempted to use security best practices such as using a secure host and server, security protocols, reCAPTCHA, reducing log-in attempts, disabling XML RPC, and many more features to create a safe and secure environment for the users.

RAMP is developed with a limited number of users willing to share their ideas. Wider use of RAMP may reveal other limitations.

RAMP neither substitutes or replaces medical advice by professionals nor provides emergency services during times of crisis. RAMP is designed for adults and is not yet adapted for adolescents and young adults.

### Conclusions

Web-based platforms such as RAMP can be powerful assets to address significant gaps in the mental health and substance use care systems and can address a major public health crisis. It can integrate different settings of care from prevention to crisis management, all on one accessible platform. In terms of its architecture, RAMP uses a modular design that allows for maximal personalization and integration of user engagement strategies. WordPress components that are used to develop RAMP are mapped onto a 3-tier architecture, where the presentation, application, and database layers communicate to ensure proper functionality for users.

Although web applications seem to be an appropriate development technology for the current and future developments of RAMP, users may also benefit from having certain features such as “Assessments” and “Goals” on a mobile app. This would allow users to access these features even without a reliable internet connection and to receive on-screen notifications as appropriate.

RAMP is currently focused on substance use disorders and specifically reducing overdose, but the tool is flexible enough to include many more domains such as suicide, anxiety, self-harm, and trauma. Broader implementation will support this concept and lead to the next level of functionality. RAMP’s modular design makes it a promising tool for addressing a breadth of mental health conditions among vulnerable populations, increasing the capacity of clinical settings and reducing barriers to care. Further research will need to investigate whether this architecture style is appropriate for eHealth tools addressing different populations and appropriate implementation strategies for eHealth tools addressing people who use drugs.

## Abbreviations

API: application programming interface
CMS: content management system
HRA: High-Risk Assessment
RAMP: Risk Assessment and Management Platform

## Data Availability

Data sharing is not applicable to this article as no data sets were generated or analyzed during this study.
